# The significance of post-translational removal of α-DG-N in early stage endometrial cancer development

**DOI:** 10.18632/oncotarget.17286

**Published:** 2017-04-20

**Authors:** Sophea Heng, Jemma Evans, Lois A. Salamonsen, Tom W. Jobling, Guiying Nie

**Affiliations:** ^1^ Centre for Reproductive Health, Hudson Institute of Medical Research, Clayton, Victoria, Australia; ^2^ Department of Molecular and Translational Sciences, Monash University, Clayton, Victoria, Australia; ^3^ Department of Biochemistry and Molecular Biology, Monash University, Clayton, Victoria, Australia; ^4^ Department of Obstetrics and Gynaecology, Monash University, Clayton, Victoria, Australia; ^5^ Epworth Research Institute, Epworth Health Care, Richmond, Victoria, Australia

**Keywords:** endometrial cancer, dystroglycan, tight junction, cell polarity, estrogen

## Abstract

Endometrial cancer is one of the most common gynecological malignancies affecting post-menopausal women, yet the underlying mechanisms are not well understood. Dystroglycan (DG) is a large glycoprotein, consisting of α- and β-subunits that are non-covalently associated with each other. Modifications to α-DG have been linked to a variety of cancers, where the N-terminus of α-DG (α-DG-N) is post-translationally removed by a furin-like enzyme. However, the functional significance of α-DG-N removal is unknown. Our previous studies have established that the α-DG cleavage enzyme furin is significantly up-regulated in endometrial cancer. This study aimed to investigate the importance of α-DG-N removal in post-menopausal endometrial cancer. We demonstrated that α-DG-N removal predominantly occurred in early stage endometrial cancer tissues, and that the cleaved α-DG-N was significantly elevated in the uterine lavage of early grade endometrial cancer patients. Furthermore, α-DG-N removal significantly decreased the tight junction integrity and polarity of the endometrial epithelial cells, promoting the loss of polarity markers scribble and atypical protein kinase C (aPKC) and reducing the trans-epithelial electrical resistance. The removal of α-DG-N also sensitized the cells for estrogen-dependent proliferation. These results strongly suggest that α-DG-N removal plays an important role in early stage development of endometrial cancer, and that the elevated levels of α-DG-N in uterine fluid may provide a biomarker for early detection of endometrial cancer.

## INTRODUCTION

Endometrial cancer is one of the most common gynecological malignancies [1], predominantly affecting women in their post-menopausal years. However, recent studies show that up to 30% of women diagnosed with endometrial cancer are pre-menopausal and this incidence is increasing in line with the increasing prevalence of obesity [2].

According to the International Federation of Gynecology and Obstetrics, endometrial cancer is graded as follows: grade 1, tumor is well differentiated with 5% solid growth pattern; grade 2, tumor is moderately differentiated with 5–50% solid growth pattern; and grade 3, tumor is poorly differentiated with 50% solid non-morular pattern [3–5]. Based on clinical, endocrine and epidemiological characteristics, endometrial cancers are also classified as either type I or type II [6, 7]. Type I endometrial tumors are estrogen dependent, associated with endometrial hyperplasia and have favorable outcomes [6–8]. Type P tumors are estrogen independent, associated with endometrial atrophy and have less favorable outcomes [6–8].

Diagnosing endometrial cancer at early and confined stages is often problematic for cycling women where no obvious symptoms are detected, whilst symptoms such as vaginal bleeding in post-menopausal women are an indicator that the cancer has already progressed. The most common effective treatment following such diagnosis is abdominal hysterectomy (total removal of the uterus). Identifying novel molecules and pathways associated with early stage endometrial cancer is important for early diagnosis and non-surgical management. Minimally invasive tests that can be performed at routine gynecological checkup are currently non-existent.

Early changes in cellular function for cancer development include the loss of tight junction integrity, apical-basal polarity and cell-to-cell contacts. These are strong indicators that the cells are losing polarity and entering into an epithelial-mesenchymal transition (EMT), a key phenotype of early cancer progression [9–12]. The hallmark of EMT involves cellular loss of epithelial-specific adherens and tight junction proteins such as E-cadherin and occludin in association with loss of cell-cell contact and cell polarity [13, 14]. Furthermore, disruption in apical-basal epithelial polarity affects three major protein complexes, the PAR complex, located on the apical junction domain of the epithelial cell, is and important for the formation of the tight junction [15, 16]; the crumbs complex, localized to the apical membrane for interacting with the PAR complex [17, 18]; and the scribble complex, localized in the basolateral domain of the epithelial cells [19, 20].

Dystroglycan (DG), a large cell surface glycoprotein, has been linked to a number of cancers [21–26]. However, its role in human endometrial cancer remains largely unknown. DG is ubiquitously expressed in epithelial cells, and plays an important role in cell adhesion as a linker between the extracellular matrix and the cytoskeleton. DG, encoded by the DAG1 gene [27], is initially synthesized as one precursor protein, which is then post-translationally cleaved into two non-covalently associated α- and β-subunits [28]. The β-DG subunit is anchored within the plasma membrane, whereas α-DG is non-covalently associated with the extracellular N-terminus of β-DG. The α-DG subunit contains a central mucin-like region [29, 30], which is heavily glycosylated [31, 32] and mediates cell adhesion. However, the central region of α-DG is obstructed by its large N-terminus (α-DG-N, amino acid 30–312). Proteolytic cleavage at amino acid 312 removes α-DG-N [30, 33, 34], exposing the central region of α-DG to interact with extracellular proteins such as laminin and fibronectin [30, 35]. The function of α-DG is also dependent on glycosylation [36]. Mutations in glycosyltranferase genes that impact on α-DG glycosylation lead to hypo-glycosylation of α-DG, reducing its laminin binding activity [37]. DG also plays important roles in the formation of the basement membrane, as loss of DG has been linked to various epithelial cancers’ progression [21–25, 38]. Loss of α-DG expression correlates with high grades of malignancies and reduced survival rate in breast and prostate [21, 22], retina [23, 26], colon and gastric [24], cervix and vulvar cancer patients [25]. Loss of α-DG in cancer cell lines leads to loss of extracellular binding; this can result from alterations in α-DG glycosylation or proteolytic cleavage of α-DG by furin [39].

Furin is a member of the proprotein convertase (PC) family of serine proteases. It post-translationally cleaves precursor proteins at the motif of (K/R-(X)n-, X is any other amino acid) [40]. Furin is ubiquitously expressed and has a number of important functional roles in cancer [41]. We have previously demonstrated that furin is the only PC member that is significantly up-regulated in post-menopausal endometrial cancer tissues [4]. Consistent with this, total PC activity is also significantly increased in uterine lavage of endometrial cancer patients compared to control post-menopausal women [4]. Previous studies have reported that furin plays a direct role in the cleavage and removal of α-DG-N in breast cancer cells, and that this is inhibited by a PC inhibitor [39]. Furthermore, cleavage of α-DG-N at the predicted PC cleavage site (amino acid 312) has been confirmed with a purified GST-α-DG-N antibody [33].

To date, little is known about the significance of α-DG-N removal in cancer development. These present study investigated the importance of α-DG-N removal in influencing endometrial epithelial cell characteristics relevant to cancer development.

## RESULTS

### Immunolocalization of α-DG in human endometrial cancer tissues

**Figure 1:**
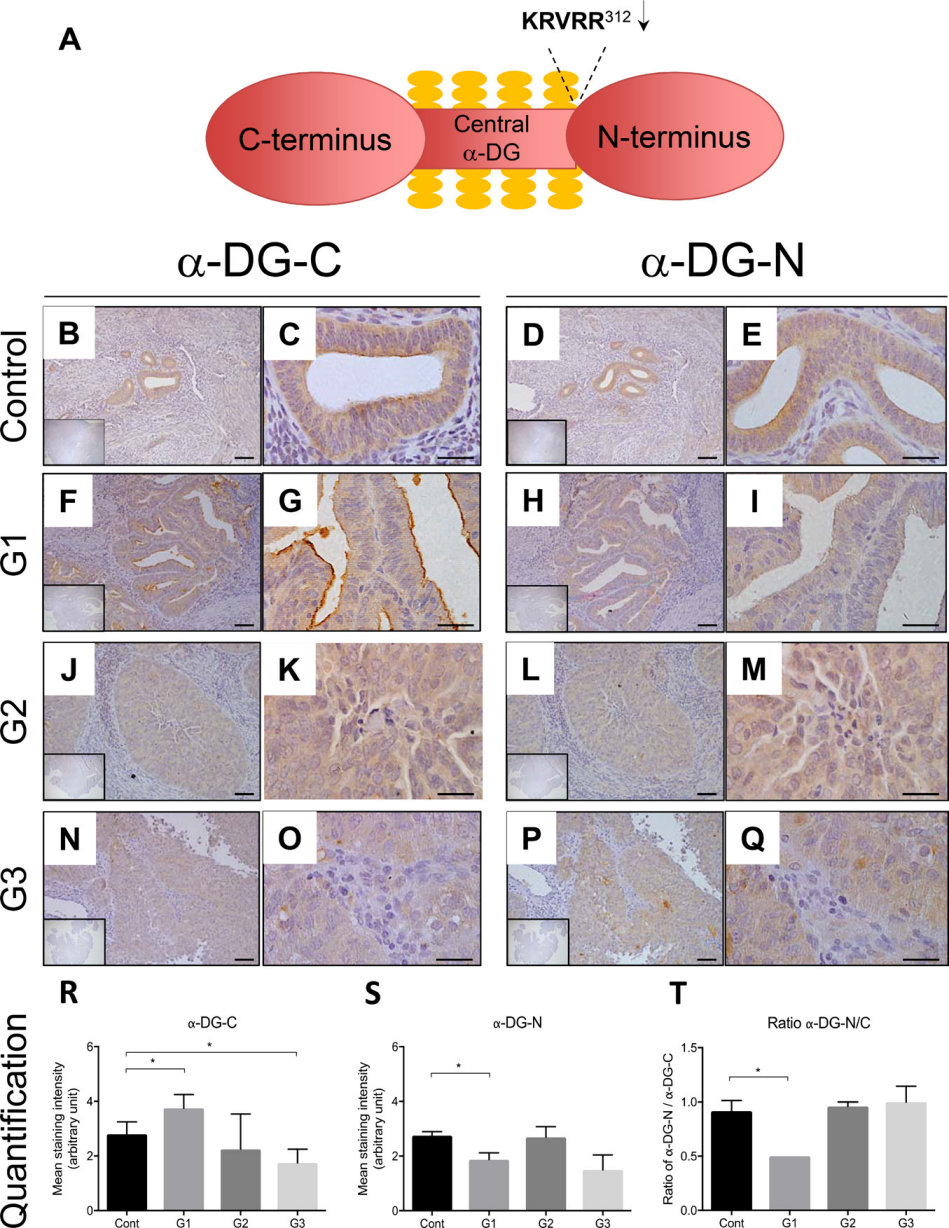
Representative images of immunolocalization of α-DG in the endometrium of post-menopausal women without (control) and with endometrial cancer Schematic diagram of α-DG protein structure (Figure 1**A**). Serial sections were stained for α-DG-C and α-DG-N respectively for control (**B**–**E**), grade 1 (**F**–**I**), grade 2 (**J**–**M**), and grade 3 cancer sections (**N**–**Q**). The inserts are negative controls. (**R**) and (**S**) Mean intensity of glandular epithelial staining for α-DG-C and α-DG-N respectively. (**T**) The ratio of α-DG-N/α-DG-C. Each bar represents mean ± SD (*n =* 4–8 for each phase), *p < 0.05.

To assess the physiological relevance of α-DG-N removal in endometrial cancer, we immunostained the C- and N-termini of α-DG (α-DG-C and α-DG-N respectively) (Figure 1A) in tissues from post-menopausal women without (controls) and with different grades of endometrial cancer (grade 1, 2 and 3, *n =* 4–8 per group). The α-DG-C was immunolocalized to glandular epithelial cells in all endometrial cancer and control tissues that were examined (Figure 1B–1C, 1F–1G, 1J–1K and 1N–1O). Similar localization was observed for α-DG-N (Figure 1D–1E, 1H–1I, 1L–1M, 1P–1Q). Compared to controls (Figure 1B–1C, 1R), the staining intensity of α-DG-C was significantly elevated in grade 1 cancer (Figure 1F–1G, 1R), showed no difference in grade 2 (Figure 1J–1K, 1R), but was significantly reduced in grade 3 cancer (Figure 1N–1O, 1R). These data demonstrate that α-DG-C immunostaining intensity decreases with increasing grade of cancer. In contrast, the staining intensity of α-DG-N was significantly reduced in grade 1 cancer (Figure 1H–1I, 1S), but not significantly altered in grade 2 or 3 cancer (Figure 1L–1M and 1P–1Q respectively, and 1S) compared to controls (Figure 1D–1E, 1S).

If α-DG-N is removed, a reduction in the ratio of α-DG-N over α-DG-C would be expected. The α-DG-N/α-DG-C ratio was less than one in grade 1 cancer but close to one in controls or grades 2 or 3 cancer (Figure 1T), consistent with α-DG-N being post-translationally removed from the epithelial cells in grade 1 endometrial cancer. These results suggest that post-translational removal of α-DG-N from the epithelial cells may be associated with early stage endometrial cancer development.

### Detection of α-DG-N in uterine fluid from endometrial cancer patients

**Figure 2:**
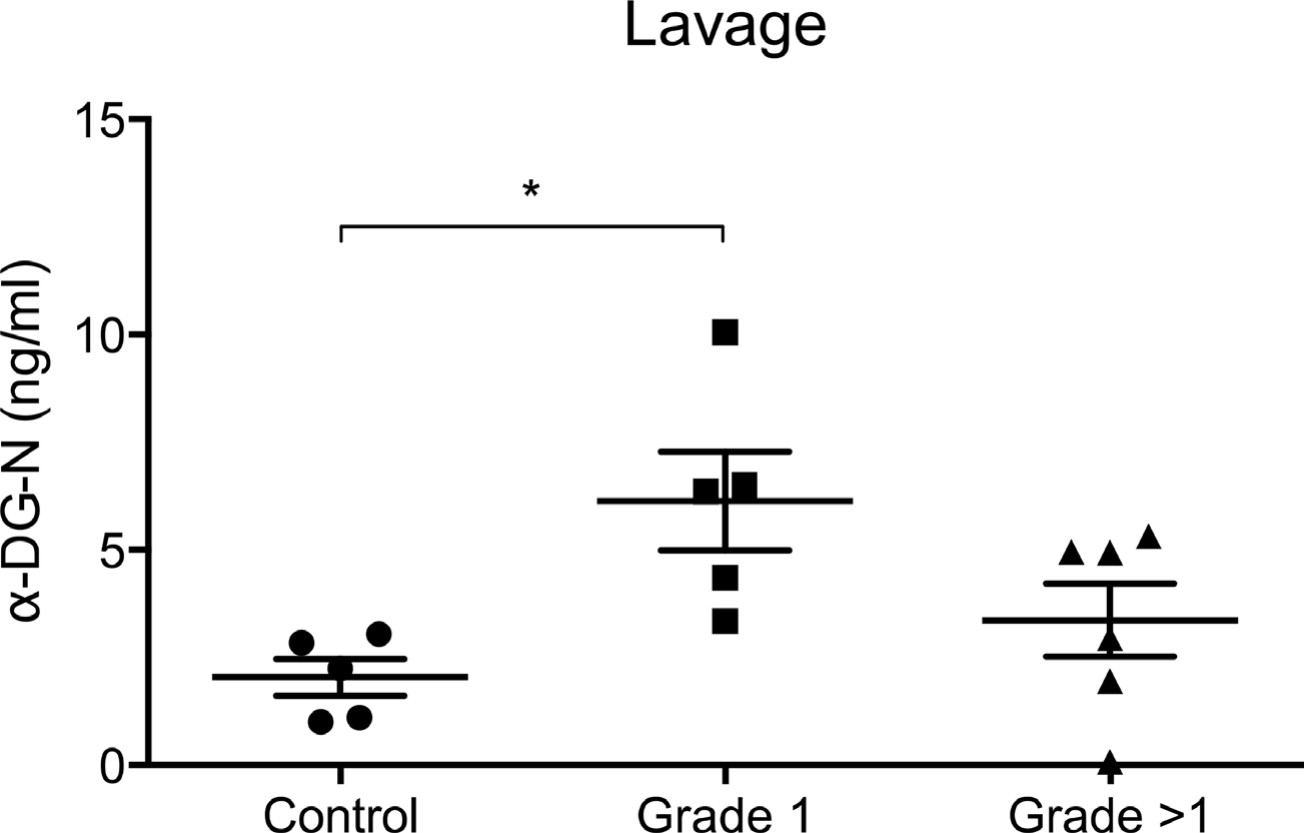
ELISA detection of α-DG-N in the uterine lavage Significantly higher concentrations of α-DG-N were detected in grade 1 endometrial cancer compared to control post-menopausal women, *p < 0.05.

We next assessed whether the α-DG-N cleaved from endometrial tissue is detectable in uterine lavage and whether these levels are altered in endometrial cancer patients. ELISA assessment of uterine lavage demonstrated the presence of α-DG-N in all samples examined (Figure 2). The concentration of α-DG-N in the lavage was significantly higher in women with grade 1 cancer than controls (Figure 2). This difference was not observed in > grade 1 endometrial cancer (Figure 2). This result is consistent with more α-DG-N being cleaved from the endometrial surface in grade 1 cancer and accumulating within the uterine fluid, confirming the immunostaining data presented in Figure 1.

### Removal of α-DG-N promotes the loss of polarity in endometrial epithelial cells

**Figure 3:**
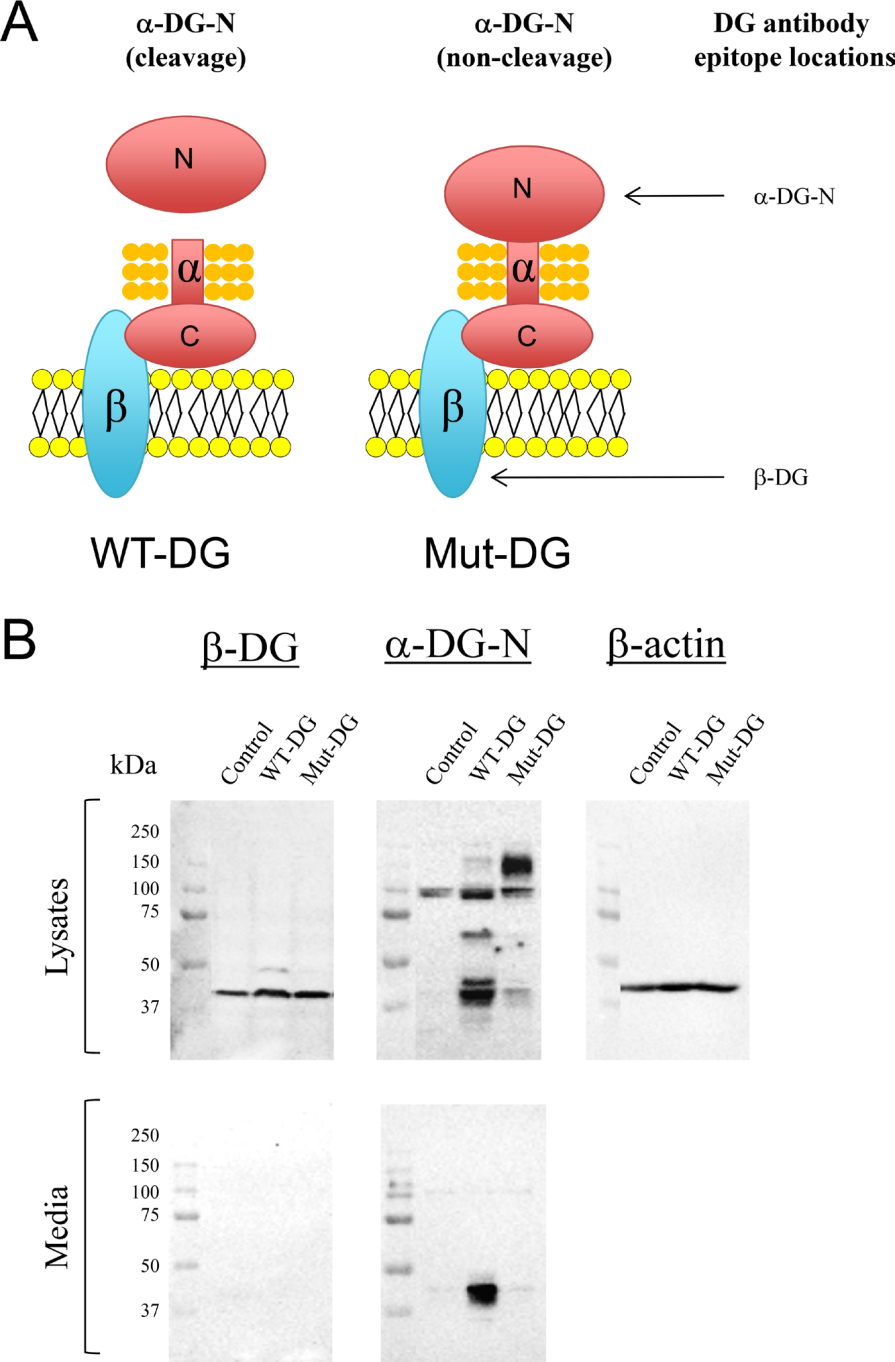
Western blot analysis of DG subunits in control, wild type (WT) and mutant (Mut) DG stable Ishikawa cell lines (**A**) Schematic illustrations of the difference between WT and Mut-DG and the epitopes of two DG antibodies. (**B**) Western detection with the two antibodies shown in (A) in the lysates and media of control, WT-DG and Mut-DG cells. Equal loading was confirmed by western analysis of β-actin in the lysates.

To investigate the importance of α-DG-N removal in early stage endometrial cancer development, we utilized our previously established Ishikawa cell lines stably expressing wild-type-DG (WT-DG, which contains the furin-cleavage site) and mutant-DG (Mut-DG, in which the PC-cleavage site was mutated to prevent α-DG-N cleavage) [30]. The only difference between WT-DG and Mut-DG is a single amino acid mutation at the PC cleavage site at amino acid 312 (Figure 3A). Western blot of cell lysates detected elevated levels of β-DG in both WT-DG and Mut-DG cells compared to control Ishikawa cells (Figure 3B), confirming overexpression of DG in both WT-DG and Mut-DG cells. No β-DG was detected in the media as expected (Figure 3B), since β-DG is a membrane subunit of the DG complex. However, α-DG-N showed a different pattern between WT-DG and Mut-DG cells. A strong dominant band of approximately 37 kDa, consistent with the size of the cleaved α-DG-N, was detected in both the lysates and media of WT-DG cells (Figure 3B). In contrast, Mut-DG cell media showed no band, but the lysates displayed an extra band of approximately 150 kDa that is consistent with the intact α-DG (Figure 3B). These data validated that in Mut-DG cells the cleavage and release of α-DG-N was prevented.

**Figure 4:**
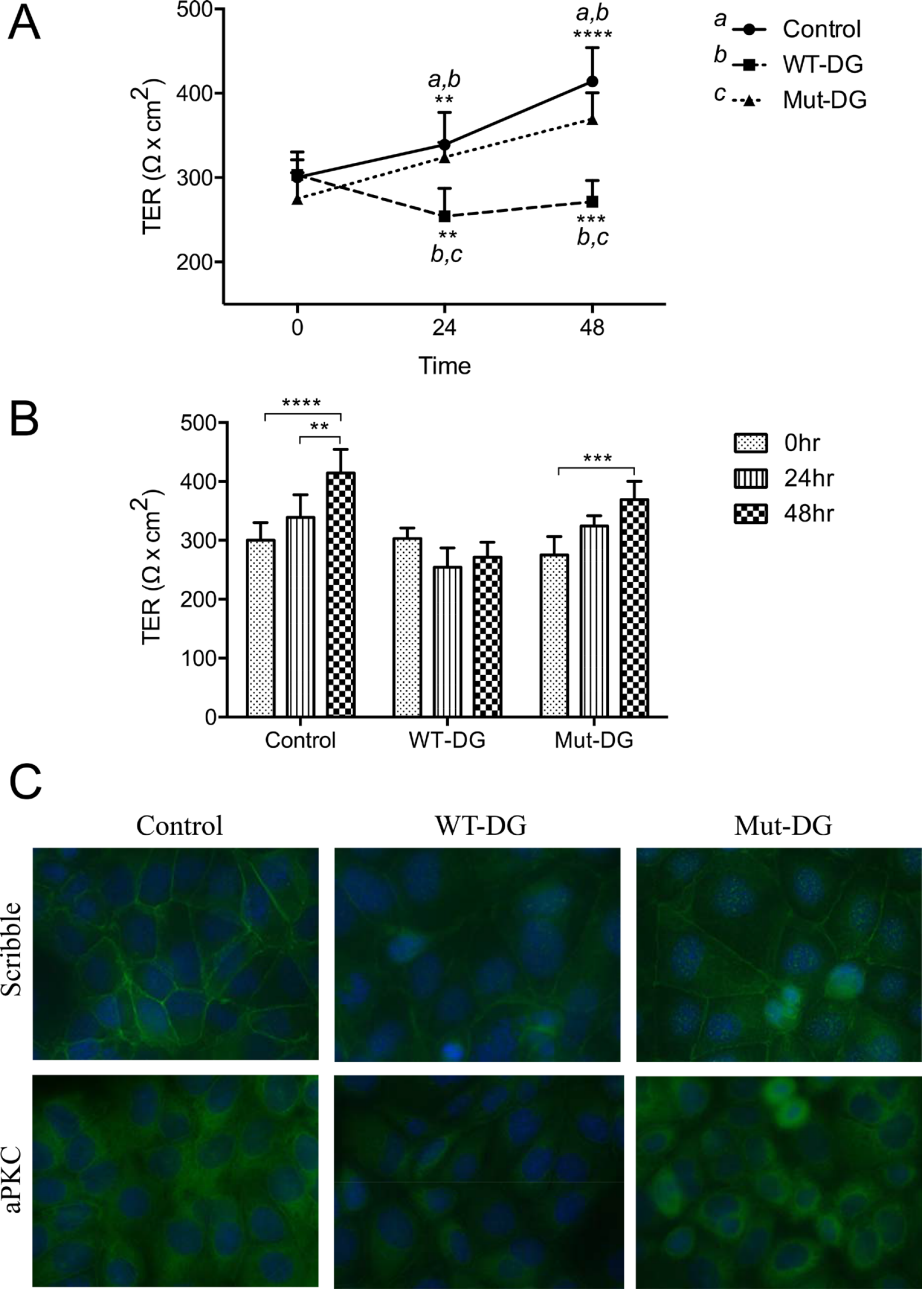
Trans-epithelial electrical resistance (TER) and tight junction integrity in different DG stable cell lines (**A**–**B**) TER of control, WT-DG and Mut-DG cells at 0, 24 and 48 hr of culture. Data presented are mean ± SD (*n =* 4), **p < 0.005, ***p < 0.0005 and ****p < 0.0001. (**C**) Immunofluorescence staining for cell polarity marker proteins scribble and aPKC in green. Nuclei were stained for DAPI in blue.

As loss of epithelial cell apical-basal polarity is a hallmark of early stage cancer development, we next determined whether cleavage of α-DG-N was important for cell polarity and monolayer integrity. Trans-epithelial electrical resistance (TER) was measured as a proxy readout of polarity in the DG-manipulated Ishikawa cells. Each cell line was grown to a confluent monolayer on transwell inserts, and TER readings were then taken every 24 hr for 48 hr. All three cell lines showed a very similar TER at the start of the experiment (Figure 4A–4B). Over 48 hr of culture, TER progressively increased in the control cells, consistent with the establishment of a continuous intact monolayer with apical-basal polarity. Cells expressing the mutated DG (Mut-DG) likewise increased TER progressively over time (Figure 4A–4B). In contrast, in WT-DG cells, which release α-DG-N, TER decreased over time (Figure 4A–1B), indicating that overexpression of WT-DG with the associated cleavage of α-DG-N promoted the progressive loss of apical-basal polarity. These results suggested that α-DG-N removal influences α-DG function and the loss of cellular apical-basal polarity.

We further examined the apical-basal polarity markers scribble and atypical Protein Kinase C (aPKC) by immunofluorescence in these cell lines (Figure 4C). Membrane localization of scribble was seen in all cell lines. However, the staining intensity was greatly reduced in WT-DG cells versus controls (Figure 4C), while Mut-DG cells were not different from controls (Figure 4C). Immunostaining for aPKC showed a similar pattern to scribble (Figure 4C), again the staining was reduced in WT-DG but not in Mut-DG cells or controls. Because of this observation, we immunostained aPKC in the tissues investigated in Figure 1. The staining intensity of aPKC was significantly lower in grade 1 cancer tissues compared to the controls (Supplementary Figure 1). These results further confirmed that α-DG-N cleavage affected the presentation of polarity markers scribble and aPKC, consistent with α-DG-N removal regulating the apical-basal epithelial polarity to promote depolarization, which is a key phenotype in early cancer progression.

### Cleavage of α-DG-N promotes estrogen-dependent cell proliferation

**Figure 5:**
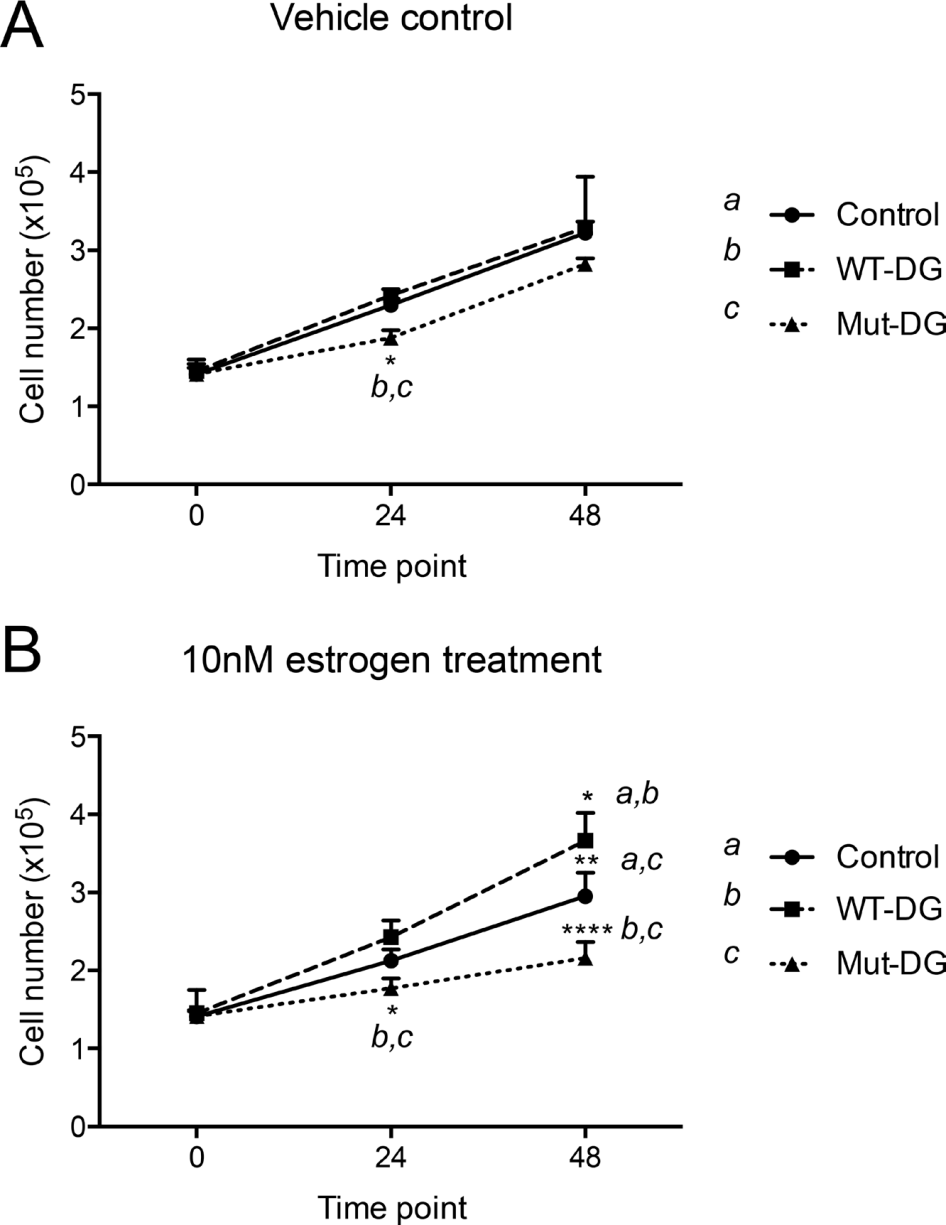
Proliferation of DG stable cells in the absence and presence of estrogen (**A**) Cells in vehicle control for 0, 24 and 48 hr, *p < 0.05. (**B**) Cells in 10 nM estrogen, *p < 0.05, **p < 0.005 and ****p < 0.0001.

As elevated levels of estrogen in post-menopausal women are a major risk factor for endometrial cancer development [42], we next assessed whether cleavage of α-DG-N affects estrogen-dependent cell proliferation. When treated with vehicle control (ethanol only), cell proliferation was similar between control and WT-DG but reduced in Mut-DG, so that the proliferation rate of WT-DG cells was significantly greater than Mut-DG cells at 24 hr, but not 48 hr (Figure 5A). In the presence of 10 nM estrogen, compared to control cells, the WT-DG cells proliferated significantly faster, whereas the Mut-DG cells proliferated significantly slower (Figure 5B). These differences were obvious at 24 hr and became significant at 48 hr (Figure 5B). As the only difference between the WT-DG and Mut-DG is the presence or absence of α-DG-N cleavage, these results provide evidence that α-DG-N removal from the cell surface promotes estrogen-dependent endometrial epithelial cell proliferation.

## DISCUSSION

The mechanisms underlying endometrial cancer development, especially during the early stages of the disease are poorly understood. This study demonstrated that post-translational modification of DG may play an important role in early stage development of endometrial cancer. DG mediates cell adhesion through the central glycosylated region of α-DG. However, this region is obstructed by its large N-terminus (α-DG-N), which is known to be removed from the cell surface by a furin-like PC enzyme in cancer [39]. We previously reported that furin is the only PC that is up-regulated in endometrial cancer in post-menopausal women [4]. In this study, we proved by immunolocalization that α-DG-N was removed from the endometrial epithelial cells of grade 1 cancer, and that α-DG-N levels in uterine fluids were significantly elevated in grade 1 endometrial cancer patients.

The significance of α-DG-N removal in early stage endometrial epithelial cancer was further investigated by utilizing Ishikawa cells stably expressing different forms of α-DG [30]. WT-DG cells express α-DG that contains the furin-cleavage site, and release α-DG-N, while the Mut-DG cells express α-DG that lacks the furin-cleavage site and hence α-DG-N is retained on the cell surface. Trans-epithelial electrical resistance measurement demonstrated that the removal of α-DG-N profoundly decreased tight junction integrity, in that WT-DG cells were significantly less polarized than the control (empty vector) or Mut-DG cells. Scribble, one of the key protein responsible for establishing apical-basal cell polarity binds to proteins such as E-cadherin to further stabilize cell adhesion [19]. Additionally, scribble is a potential tumor suppressor as its down-regulation is often associated with cancer [43, 44]. The aPKC protein is part of the PAR complex, which is localized at the apical side of the tight junction and functions to promote the formation of tight junctions [16, 45]. In this study, the removal of α-DG-N reduced the presentation of scribble and aPKC in WT-DG compared to control or Mut-DG cells, providing a mechanism underpinning the disruption of tight junction integrity and apical-basal polarity, promoting cell depolarization.

The loss of tight junction integrity is a strong indication that the cells are undergoing an epithelial-mesenchymal transition (EMT), a key phenotype in early cancer progression [9, 11, 46, 47]. Destabilization of cell-cell adherens junctions and tight junctions are important for cells to enter into EMT. The transition of polarized epithelial cells into a plastic and motile mesenchymal phenotype is initially due to the breakdown of tight junction integrity and disruption in three major protein complexes (PAR, crumbs and scribble) that are important for apical-basal cell polarity [9, 46]. This is followed by the weakening and breakdown of adherens junctions and desmosomes [46] through post-translational modifications of proteins such as E-cadherin and β-catenin [48, 49] and transcriptional repression such as zinc finger E-box-binding homeobox (ZEB) and Snail [50–53]. In this study, the removal of α-DG-N from the cell surface led to disruption of scribble and the PAR complex protein aPKC. Further studies are required to investigate how α-DG-N removal influences these changes.

Elevated levels of estrogen are associated with endometrial cancer development in post-menopausal women [42], with the main source of estrogen production being adipose tissues. Elevated estrogen production from adipose tissues, in obesity and the use of hormone replacement therapy are linked to cancer development in such women [54, 55]. In this study, α-DG-N removal significantly promoted estrogen-dependent proliferation of endometrial epithelial cells.

In summary, we have demonstrated that the removal of α-DG-N plays an important role during early stage development of endometrial cancer. The removal of α-DG-N decreases tight junction integrity and cell polarity, which are directly related to epithelial-mesenchymal transition. The removal of α-DG-N also sensitizes the cells for estrogen-dependent cell proliferation. The molecular mechanisms governing these effects will need to be further investigated. Furthermore, α-DG-N cleaved from the endometrial epithelium is released into the uterine cavity. Thus elevated α-DG-N in uterine fluid may provide a potential biomarker for non-invasive detection of early stage endometrial cancer in post-menopausal women.

## MATERIALS AND METHODS

### Clinical material collection

Ethical approval was obtained from the Human Ethics Committee at Monash Medical Centre (Melbourne, Australia) and written informed consent was provided by individual patient. Endometrial biopsies from post-menopausal control (*n =* 5) and endometrial cancer patients (*n =* 16) were fixed in formalin and embedded in wax as previously described [3]. Endometrial cancer grade (G) (G1, *n =* 4; G2, *n =* 8 and G3, *n =* 4) were confirmed by histological grading [3]. Uterine lavages (*n =* 16) were obtained from women with (*n =* 11) or without (*n =* 5) endometrial cancer. Tissues and lavages were collected from different patients for this study. In brief, 3 ml of sterile saline was gently infused transcervically through a fine flexible catheter into the uterine cavity, and the uterine fluid was recovered by aspiration. Cell debris was removed by centrifugation for 5 min at 1000 rpm and aliquots were stored at –80°C.

### Immunohistochemical localization of α-DG and aPKC in human endometrial tissues

Sections of µM were de-paraffinized in histosol, rehydrated and microwaved for 10 min in 0.01 mol/L citrate buffer (pH 6.0) for antigen retrieval. Endogenous peroxidase was quenched with 3% H_2_O_2_ in methanol for 10 min and non-specific binding was blocked with pre-immune serum. The sections were incubated for 1 hr at 37°C with primary antibodies for α-DG-C (C15 at 5 μg/ml, Santa Cruz Inc., California, USA) and aPKC (at 1 μg/ml, Santa Cruz Inc., California, USA) or overnight at 4°C for α-DG-N (WH00 at 6 μg/ml, Sigma Aldrich, NSW, Australia). Mouse or goat IgG (Dako, NSW, AUS) replaced the primary antibodies as the respective negative controls. Sections were washed and appropriate biotinylated secondary antibodies (Vector laboratories, Inc. USA) were applied for 30 min at room temperature. Signals were amplified with StreptABC/HRP (Dako) for 30 min at room temperature and visualized with diaminobenzidine (Dako). The relative immunostaining intensity in epithelial cells within the entire tissue section was evaluated and scored semi-quantitatively by two independent observers (0 = no staining, 4 = maximal staining). The data was expressed as the mean intensity ± SD.

### ELISA detection of α-DG-N in human uterine lavage of endometrial cancer patients

A previously established ELISA for the detection of α-DG-N in uterine lavage was used [56]. In brief, half area 96-well plates were coated overnight at 4°C with 0.5 μg/ml of α-DG-N mAb (2A3, Sigma Aldrich) in 0.1 M sodium carbonate/bicarbonate buffer of pH 9.6 (Sigma Aldrich). The wells were then washed with PBS (137 mM NaCl, 2.7 mM KCl, 10 mM Na_2_HPO_4_ and 1.8 mM KH_2_PO_4_) containing 0.05% Tween20 (PBS-T), and blocked with 1% BSA (Bovogen, VIC, Australia) in PBS for 90 min at 37°C. The wells were washed with PBS-T, incubated with recombinant α-DG-N as the standards or uterine lavage (undiluted) for 2 hr with gentle agitation. The wells were washed and incubated first with 3 μg/ml of biotinylated detection antibody (3B4, Creative Diagnostics, NY, USA) for 1 hr, then with poly streptavidin-HRP (Thermo Scientific, Waltham, MA, USA) at 1:25,000 dilution for 50 min with gentle agitation, and finally with ultra-TMB substrate (undiluted, Thermo Scientific) for 7–10 min in the dark at room temperature without agitation. The ELISA reaction was stopped with 1 M sulfuric acid (Banksia Scientific Company Pty Ltd, QLD, AUS) and the absorbance at 450 nm was measured (Envision Multilabel reader, PerkinElmer, Waltham, MA, USA). All washes and incubations were performed at room temperature.

### Culture of Ishikawa cells stably expressing different forms of α-DG

Ishikawa cells (generous gift by Professor Masato Nishida of National Hospital Organization, Kasumigaura Medical Center, Ibaraki-ken, Japan), were validated by karyotype analysis via Short Tandem Repeat (STR) DNA profiling of human cell lines per ATCC guidelines [57]. Ishikawa cells were stably transfected with constructs to express control (empty vector), wild-type (WT) or mutant (Mut) DG that lacks the PC cleavage site [30]. These DG stable cells were cultured in complete media containing Modified Eagle’s Medium (MEM, Life Technologies, Carlsbad, CA, USA) supplemented with 10% (v/v) fetal calf serum (FCS) (Life Technologies) and 2% geneticin (Sigma, USA) at 37°C. For estrogen treatment, cells were grown in complete media containing MEM supplemented with 10% (v/v) charcoal-stripped fetal calf serum (csFCS) (Life Technologies) and 2% geneticin.

### Protein extraction and Western blot analysis

DG stable cells were lysed with 50 mM Tris/HCl (pH 7.4), 150 mM NaCl, 1% (v/v) Triton X-100, 1 mM EGTA and 2 mM EDTA containing protease inhibitors (Calbiochem, Darmstadt, Germany), and proteins were collected following centrifugation (14,000 rpm for 10 min at 4°C). To collect serum-free conditioned media, cells were seeded at the same density grown to approximately 80% confluency in complete media, then washed with PBS and grown in serum-free MEM media containing 2% geneticin for a further 48 hr. Protein concentrations were determined by a Bradford assay (Bio-rad Laboratories CA, USA), and standard western blot (10% SDS-PAGE gel) was performed using primary antibodies for β-DG (4F7 at 1 μg/ml, Santa Cruz) and α-DG-N (WH00 at 1 μg/ml, Sigma Aldrich) with appropriate anti-HRP secondary antibodies (Dako, NSW, AUS). To confirm equal loading, blots were stripped and re-probed for β-actin using a HRP-conjugated antibody (0.4 μg/ml, Cell Signaling, MA, USA).

### Trans-epithelial Electrical Resistance (TER) measurement

Permeable transwell inserts (6.5 mm, 0.4 μm pore, Corning, NY, USA) were coated with 10 μg/ml fibronectin (BD Bioscience, NSW, AUS) for 1 hr at room temperature. DG stable cell lines were seeded (4 × 10^4^ cells per insert) on fibronectin-coated inserts and allowed to attach overnight in complete media. They were then incubated in serum-free media containing 2% geneticin in the upper chambers and complete media in the lower chambers. Trans-epithelial electrical resistance (TER) was measured daily using a Millipore MilliCell-Electrical Resistance System (Millipore, Massachusetts, United States), commencing from the day of media change (Day 1 or otherwise 0 hr). Cells were maintained at 37°C throughout the experiment. Following the removal of the plate from the incubator and before TER measurement, cells were equilibrated on a warming plate within the culture hood for approximately 10 min. Four TER readings (ohm per cm^2^) were taken from each well and readings from duplicate wells per cell line were averaged to obtain the raw TER; the final value was obtained after subtraction of background TER which was obtained from inserts that contained no cells in the same experiment. The entire experiments were repeated independently four times and the data was expressed as mean ± SD (*n =* 4).

### Immunofluorescence analysis

DG stable cells were grown on chamber slides, fixed in 100% methanol, and blocked with 10% goat serum, 2% human serum, 0.1% fish skin gelatin and 0.1% Triton X-100 in PBS containing 0.2% Tween20 for 1 hr at room temperature. Cells were probed first with primary antibodies for scribble (at 5 μg/ml, Abcam, MA, USA) or aPKC (at 2 μg/ml, Santa Cruz, California, USA) overnight at 4°C, then with a goat anti-rabbit biotinylated secondary antibody (Vector Laboratories, Inc. USA) for 1 hr at room temperature, and Alexa 488 (at 5 μg/ml, Invitrogen, Molecular Probes, Carlsbad, CA) for 2 hr at room temperature. The nuclei were stained with DAPI (at 0.5 mg/ml, Sigma Aldrich) and the signal was visualized by fluorescence microscopy (Olympus BX53).

### Cell proliferation and estrogen treatment

DG cell lines were first cultured in 12-well plates (1 × 10^5^ cells per well) for 24 hr in complete media containing 10% csFCS, then changed to fresh complete media containing vehicle control (VC, ethanol only) or 10 nM estrogen (E) (17β-estradiol, Sigma, USA, diluted in ethanol), and cultured for 24 hr or 48 hr. Cells were trypsinized and counted using the Countess (Invitrogen, Carlsbad, CA, USA) to determine cell numbers before and after (24 and 48 hr) the treatment. The entire experiment was repeated independently 4 times (*n =* 4).

### Statistics

Data were expressed as mean ± SD. Statistical analysis was performed on raw data; comparisons between two groups used unpaired, non-parametric, Mann-Whitney test, multiple group comparisons used one-way ANOVA, non-parametric and Kruskal Wallis test (PRISM version 6.00, GraphPad Software, San Diego, CA). *p < 0.05 was taken as significant; **p < 0.005, ***p < 0.0005 and ****p < 0.0001 were considered highly significant.

## SUPPLEMENTARY MATERIALS FIGURE


