# Supplementary material for: The significance of post-translational removal of α-DG-N in early stage endometrial cancer development
Source: Oncotarget. 2017 Apr 20;8(47):81942–52. doi: 10.18632/oncotarget.17286 (PMC5669861; doi:10.18632/oncotarget.17286)
Supplement: Supplementary file 1 [file oncotarget-08-81942-s001.pdf]

The significance of post-translational removal of α-DG-N in early stage endometrial cancer development

Supplementary Materials

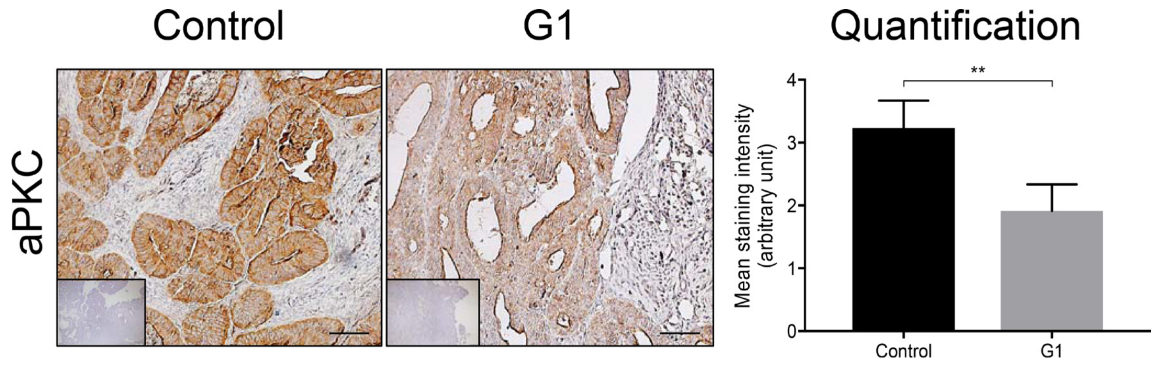

Supplementary Figure 1: Representative images of immunostaining of aPKC in the endometrium of post-menopausal women without (control) and with grade 1 (G1) cancer. Inserts are negative staining controls. Mean intensity of glandular epithelial staining for aPKC was quantified and data are presented as mean ± SD (control, $n = 5$; G1, $n = 4$), $^{**}p < 0.005$. Bar = 50 µM.
